# Conformation and membrane interaction studies of the potent antimicrobial and anticancer peptide palustrin-Ca

**DOI:** 10.1038/s41598-021-01769-3

**Published:** 2021-11-17

**Authors:** Patrick B. Timmons, Chandralal M. Hewage

**Affiliations:** UCD School of Biomolecular and Biomedical Science, UCD Centre for Synthesis and Chemical Biology, UCD Conway Institute, University College Dublin, Dublin 4, Ireland

**Keywords:** Solution-state NMR, Molecular modelling

## Abstract

Palustrin-Ca (GFLDIIKDTGKEFAVKILNNLKCKLAGGCPP) is a host defence peptide with potent antimicrobial and anticancer activities, first isolated from the skin of the American bullfrog *Lithobates catesbeianus*. The peptide is 31 amino acid residues long, cationic and amphipathic. Two-dimensional NMR spectroscopy was employed to characterise its three-dimensional structure in a 50/50% water/2,2,2-trifluoroethanol-$d_{3}$ mixture. The structure is defined by an $\alpha$-helix that spans between Ile$^{6}$-Ala$^{26}$, and a cyclic disulfide-bridged domain at the C-terminal end of the peptide sequence, between residues 23 and 29. A molecular dynamics simulation was employed to model the peptide’s interactions with sodium dodecyl sulfate micelles, a widely used bacterial membrane-mimicking environment. Throughout the simulation, the peptide was found to maintain its $\alpha$-helical conformation between residues Ile$^{6}$-Ala$^{26}$, while adopting a position parallel to the surface to micelle, which is energetically-favourable due to many hydrophobic and electrostatic contacts with the micelle.

## Introduction

Host defence peptides (HDPs) are naturally occurring molecules that are secreted as part of the non-specific response of the innate immune system^1^. These molecules contribute to the immune system’s antimicrobial response either by exerting direct antimicrobial activity, or by altering the host’s immune response. HDPs are particularly prevalent in amphibians, with approximately one-third of all known HDPs having been originally isolated from amphibians. Nonetheless, HDPs are a highly conserved part of the immune response, and have been found in all species of organisms, from humans to bacteria^2–5^.

As the number of documented cases of antimicrobial resistance to conventional small-molecule drugs continues to rise, interest in antimicrobial peptides (AMPs), which can exert their antimicrobial activity against both Gram-positive bacteria and Gram-negative bacteria without the development of resistance, continues to grow^6^. AMPs possess a number of additional advantages. They are more efficacious, selective and specific than small molecules, and are degraded to naturally occurring amino acids, which reduces the danger of unfavourable drug-drug interactions. AMPs are suitable for use both in combination with conventional drugs, and as standalone substitutes. The biological activities of HDPs, however, are not limited to antimicrobial activity. HDPs have also been demonstrated to possess antifungal, antiparasitic, antiviral and anticancer activities^7–10^. Indeed, palustrin-Ca (GFLDIIKDTGKEFAVKILNNLKCKLAGGCPP) is reported to be the most potent known anticancer peptide, with an IC$_{50}$ of 0.951 μg/ml against human gastric cancer SGC-7901^11^.

Typically, HDPs are short, with a primary sequence length between 10 and 50 amino acid residues. Usually, HDPs are unstructured in pure aqueous solution, and only adopt a defined secondary structure upon contact with a biological membrane, or in a hydrophobic environment. The induced structures are predominantly $\alpha$-helical in nature, although other identified structures include $\beta$-sheet, mixed $\alpha$-$\beta$ and extended structures. A common feature of HDP structures is that they are typically amphipathic in nature, with a clear separation between the peptide’s hydrophobic and polar or charged residues. Overall, HDP’s are usually positively charged, although a number of negatively charged peptides have been isolated as well^12^. Indeed, increased positive charge, up to a certain extent, has been associated with greater antimicrobial activity^13,14^. Similarly, increased peptide hydrophobicity is associated with peptide haemolytic activity, and thereby represents another key feature that can be changed to modulate peptide selectivity^15,16^. Interestingly, peptide dimerisation has emerged as a potential strategy for increasing AMP effectiveness by enhancing pore formation^17,18^.

While HDPs are typically positively charged, anionic molecules such as lipopolysaccharides (LPS), phospholipids and teichoic acids are prevalent in prokaryotic cell membranes, which enables attractive electrostatic interactions between HDPs and their target bacterial cell membranes^19,20^. While peptide cationicity is generally a requirement for attraction to membranes, the loss of N-terminal positive charge has been shown to allow a deeper peptide insertion into the lipid bilayer^21^, although a separate study has found that reduced N-terminal cationicity results in reduced antimicrobial activity^22^. Similarly to prokaryotic membranes, the cell membranes of cancer cells are enriched in negative charge, unlike non-cancerous cell membranes which are neutral, which allows anticancer peptides to be selectively electrostatically attracted to cancer cells.

HDPs usually exert their bactericidal activity via disruption of the bacterial cell membrane, for which three main mechanisms of action have been described: the toroidal pore model, barrel-stave model and carpet model. All three membranolytic mechanisms of action are relatively non-specific, which is the reason why few cases of bacterial resistance to HDPs have been described^23,24^. The initial step in all three mechanisms is the HDP binding of the target membrane, initially facilitated via the aforementioned electrostatic attraction, then via hydrophobic attraction. As the peptide concentration at the membrane increases, eventually a threshold concentration is reached, whereupon the peptides begin to induce changes in the membrane structure^25^. As the peptide concentration continues to increase, a second threshold concentration is reached, at which membrane lysis occurs^25^. This concentration may be determined by the organism’s capacity to repair its damaged cell membranes^26^.

Generally, membrane lysis occurs either via the pore-forming toroidal pore or barrel-stave models, or the non-pore-forming carpet model. The latter requires that the bilayer curvature is disrupted, thereby disintegrating the bacterial membrane. Under the toroidal pore model, the bacterial membrane is bent from the outer leaflet inwards, resulting in the formation of a transmembrane toroidal pore lined by the peptides and the membrane’s lipid head groups^27,28^. The barrel-stave model, meanwhile, involves the peptides forming a transmembrane pore; its lumen is lined by the peptides’ hydrophobic residues, and its exterior is composed of hydrophilic residues, which are in contact with the membrane’s lipid headgroups.

Where an amphipathic peptide structure is modified so that a residue side chain on its hydrophobic face has switched polarity, membrane lysis can also occur via a fractal rupture mechanism. This mechanism relies on apertures in the upper leaflet of the lipid bilayer being caused by chemically induced peptide-lipid interfaces, thereby exposing the membrane’s hydrophobic layer and resulting in an energetically unfavourable water-lipid interface, and ultimately in structured defects in the membrane^29^.

Interestingly, some HDPs exert their activity without disruption of the lipid bilayer. Instead, these peptides translocate across the membrane without causing lysis, and instead exert antimicrobial activity by targetting an intracellular target^30^.

Our laboratory has conducted structural studies on various amphibian HDPs, including ranatuerin-2CSa^31^, XT-7^32^, alyteserin-1c^33^, brevinin-1BYa^34^ and its analogues^35^, maximin 3^36^ and maximin 1^37^. Palustrin-Ca (GFLDIIKDTGKEFAVKILNNLKCKLAGGCPP) is a 31 amino acid residue HDP first isolated from the skin of the American bullfrog *Lithobates catesbeianus*^11^. In common with other HDPs isolated from frogs belonging to the Ranidae family, it contains a cyclic disulfide bridged domain at the C-terminal end of the peptide sequence, between residues 23 and 29. Palustrin-Ca is biologically interesting, as it is non-haemolytic, and exhibits potent broad-spectrum antibacterial activity, and as previously mentioned, very potent anticancer activity, with an IC$_{50}$ of 0.951 μg/ml against human gastric cancer SGC-7901^11^. In this work, palustrin-Ca’s conformation and structural properties in a 50% H$_{2}$O-trifluoroethanol-$d_{3}$ mixture are elucidated using NMR spectroscopy, with an ensemble of model structures being determined. Additionally, the peptide’s interactions with a membrane-mimetic are simulated using an atomistic molecular dynamics simulation.

## Materials and methods

### Materials and NMR sample preparation

The palustrin-Ca peptide (MW = 3304 g/mol, purity > 95%) was purchased from ProteoGenix (Paris). The identity and purity of the peptide were confirmed using mass spectrometry and high performance liquid chromatography (Supplementary Information). 4.5 g of the peptide was dissolved in 0.6 mL of a 50% (v/v) TFE-$d_{3}$/H_2_O solution, resulting in a final peptide concentration of 2.27 mM. 3-trimethylsilyl propionic acid (TSP) and 2,2,2-trifluoroethanol (TFE-$d_{3}$) of analytical grade were obtained from Sigma-Aldrich (Ireland).

### NMR spectroscopy

A Bruker Avance 600 NMR spectrometer with a 5 mm inverse probe head at a ^1^H resonance frequency of 600.13 MHz was used to perform a number of NMR experiments at a temperature of 298 K. One-dimensional proton, 2D phase-sensitive total correlation spectroscopy (TOCSY)^38^, nuclear Overhauser effect spectroscopy (NOESY)^39^ and natural abundance $^{1}$H-$^{13}$C and $^{1}$H-$^{15}$N heteronuclear single quantum coherence spectroscopy ($^{1}$H-$^{13}$C-HSQC) and $^{1}$H-$^{15}$N-HSQC^40^ spectra were acquired, with relaxation delays of 2.5 s, 2.0 s, 1.5 s, 1.0 s and 2.0 s, respectively, and acquisition times of 3.4 s, 340 ms, 280 ms, 170 ms and 100 ms, respectively. Mixing times of 60 ms and 200 ms were used for the TOCSY and NOESY, respectively.

The ^1^H spectral widths were 6.0 kHz, 7.2 kHz, 6.0 kHz and 9.6 kHz, and the spectra were acquired with 4, 16, 32 and 64 transients for each of the 1024, 2048, 256 and 128 t1 increments for the TOCSY, NOESY, $^{1}$H-$^{13}$C-HSQC and $^{1}$H-$^{15}$N-HSQC, respectively. The ^13^C spectral width was 21.1 kHz and the ^15^N spectral width was 10.9 kHz. All two-dimensional spectra were processed with the Bruker TopSpin program, version 4.0.6 (Bruker BioSpin, Germany), using the sine squared window function, and the ^1^H signal of TSP was used as the chemical shift reference.

### Structure calculation

The NMRFAM-SPARKY program, version 3.131^41^ was used to analyse the acquired NMR spectra and integrate the NOESY cross-peaks. The integrated peak volumes were exported, and the nuclear Overhauser effect (NOE) cross-peak intensities were calibrated using the CALIBA^42^ program, yielding a set of upper distance restraints. Protons that could not be stereospecifically assigned were treated as pseudoatoms. The set of obtained distance restraints was used as input to CYANA^43^, excluding those that represent fixed distances. A force constant of 1 kJ mol$^{-1}$ Å$^{-2}$ was used to weight the distance restraints, from which one hundred structures were generated by CYANA^44^, and submitted to 20,000 steps of simulated annealing and 20,000 of conjugate gradient minimization. The 20 structures with the lowest target function values were selected, and subjected to an additional 2000 steps of conjugate gradient energy minimization with restrained backbone atoms, using the CHARMM22 force field^45^ in NAMD, version 2.12^46^. VMD (visual molecular dynamics), version 1.9.3^47^, was used to analyse the final ensemble of 20 structures. The geometry, stereochemical quality and structural statistics of the final ensemble of structures was assessed and validated using PROCHECK^48^ and the wwPDB web service^49^.

### Molecular dynamics simulation

Structural model coordinates of an SDS micelle were obtained from Jakobtorweihen et al.^50^, and used in the construction of an SDS micelle-peptide system, which was constructed by aligning the centres of mass of the micelle and the medoid palustrin-Ca structure with VMD. To ensure that the system’s charge remained neutral, chloride ions were added as counter-ions, and finally, TIP3P water was used to solvate the system.

The system was energy minimized and equilibrated using the CHARMM22 all-atom forcefield^45,51^ within NAMD version 2.12^46^. All calculations were conducted within the NPT ensemble, using the Langevin piston Nose-Hoover method^52,53^ and periodic boundary conditions. Long-range non-bonded interactions were calculated up to a switching distance of 8.5 Å, beyond which a smooth switching function truncated the energy to a cut-off of 11 Å. The PME method^54^ was used to calculate long-range electrostatic interactions at each time step. The non-bonded interaction list was also updated every step. All bonds to hydrogen atoms were constrained using the SHAKE algorithm^55^. A 2 fs timestep was employed.

The solvated peptide-micelle system was minimized for 2000 conjugate gradient steps with peptide backbone atoms fixed, and a further 2000 steps with its $\alpha$-carbon atoms restrained. The system was then heated to a temperature of 310 K over 6000 steps using Langevin dynamics, with $\alpha$-carbon remaining restrained. The system volume was equilibrated with the Langevin piston at 1 atm over 24,000 steps, with a further 24,000 steps without restraints. Finally, the system was simulated for 57.4 ns.

The radial distribution function *g*(*r*) was used for analysing the final 22.5 ns of simulation data. The *measure gofr* command within VMD was used to calculate the *g*(*r*) function between each residue’s carbon atoms and the SDS micelle’s non-sulfate atoms. The default values 0.1 and 25.0 Å were used for $\delta r$ and max *r*, respectively.

## Results and discussion

### Conformational analysis by NMR

Two-dimensional NMR spectroscopy was used to determine the structure of palustrin-Ca in a 50:50 TFE-${d_{3}}$/water solvent mixture, which is a standard solvent mixture employed in the characterisation of peptide structures that promotes the formation of secondary structures, including $\alpha$-helices^56^. Structural studies of another ranid frog-derived HDP, brevinin-1BYa, and its analogues in TFE and SDS micelles have shown that the peptide conformations in both media are comparable^34,35^. The effects that TFE exerts on peptide structures are well-studied, having been the subject of research for approximately 60 years^57,58^.

Peptides unrestrained by disulfide bonds typically are unstructured in pure aqueous solution^59–62^, as $\alpha$-helical self-aggregation is hindered by the presence of cationic or sterically bulky residues. Furthermore, sequence enrichment in lysine, which is common among AMPs, can preclude the formation of $\beta$-sheets. Consequently, most linear cationic HDPs do not possess a defined secondary structure in pure aqueous solution^63,64^.

Nonetheless, the addition of TFE has been demonstrated to induce the formation of secondary structure by enhancing $\alpha$-helical character in peptides^65^. This has been shown in studies that systematically increase TFE concentration^34,35,59,66^.

It is generally understood that the mechanism by which peptide $\alpha$-helical character is enhanced commences with the preferential aggregation of TFE molecules around the peptide, facilitated by hydrophobic interaction between peptide’s hydrophobic sidechains and the trifluoromethyl group, which allows the TFE molecules to substitute the peptide’s hydration shell. This replacement removes alternative hydrogen-bonding partners, and results in a low dielectric environment, comparable to that experienced by peptides in biological membranes, which favours the formation of intramolecular hydrogen bonds between the backbone amide groups^67,68^.

NMR conformational studies of magainin 2 demonstrated that in a pure aqueous solution, the peptide possesses an extended random coil structure^69^. Similarly, the maximin 4 antimicrobial peptide does not have a well-defined structure in pure aqueous solution^70^. Previously, a 50% TFE concentration has been found to be appropriate for the characterisation of peptide structures, as low TFE concentrations result in poor amide chemical shift dispersion, which indicates a lack of defined secondary structure^71^.

**Figure 1:**
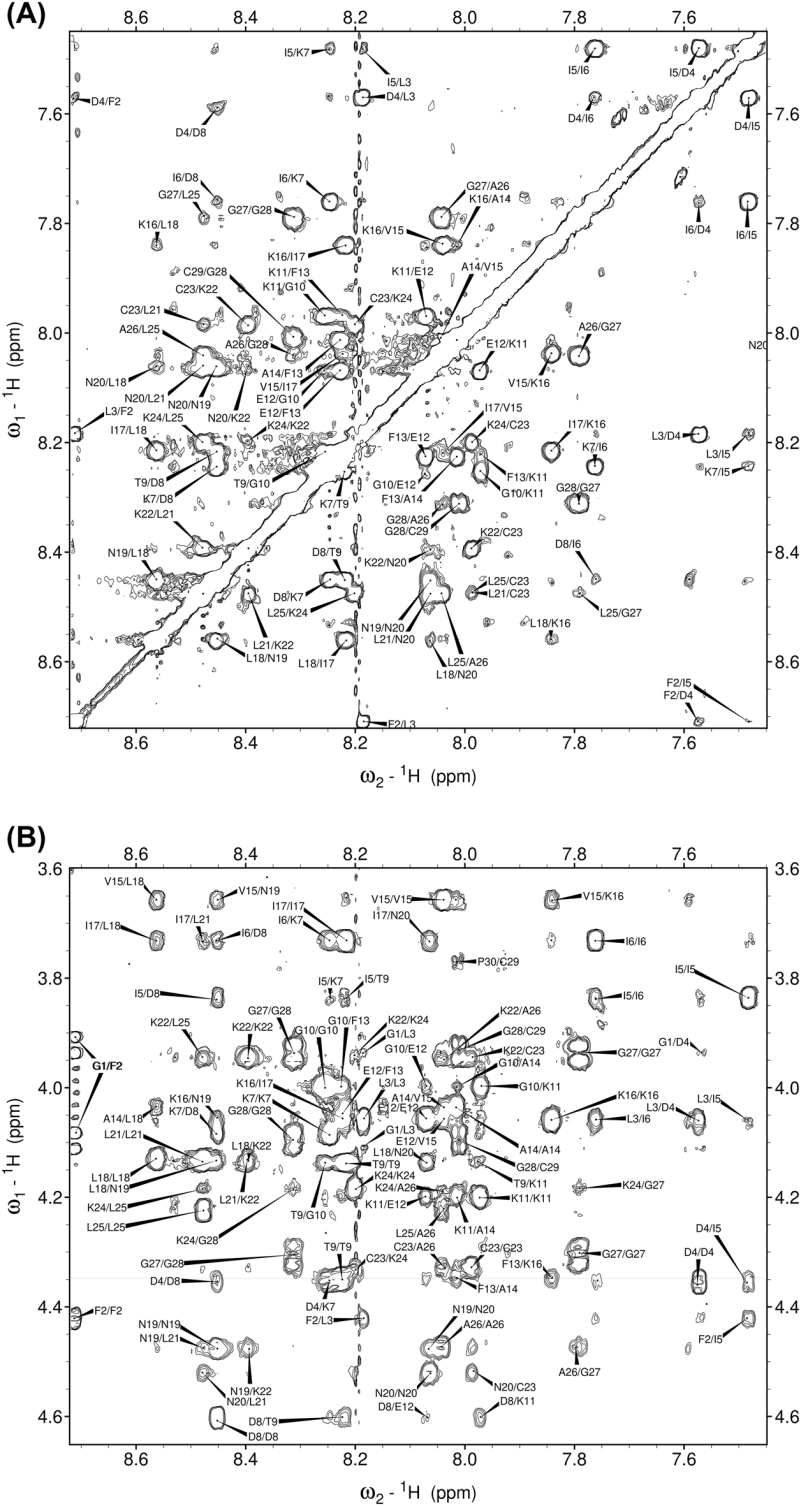
(**A**) Amide and (**B**) fingerprint regions of the 200 ms NOESY spectrum of palustrin-Ca in 50% TFE-${d_{3}}$-H$_{2}$O mixed solvent system.

The collected spectra were well-resolved, with well-dispersed peaks that facilitated unambiguous resonance assignment. Individual residue spin systems were identified with the aid of TOCSY, $^{1}$H-$^{13}$C HSQC and $^{1}$H-$^{15}$N HSQC spectra, while sequence-specific resonance assignment was conducted using the HN-HN and HN-H$\alpha$ regions of the NOESY spectrum (Fig. 1). The first sequence-specific resonance assignments were made for the unique residues Thr$^{9}$, Glu$^{12}$ and Val$^{15}$, with the remainder being assigned through the use of a backbone-walk, and investigation of HN-H$\alpha$ region peaks, where necessary. The Gly$^{1}$ amide proton chemical shift could not be determined due to chemical exchange. The presence of a disulfide bridge was confirmed by observation of clear long-range $\beta _{i}$-$\beta _{i+6}$ NOE peaks between residues Cys$^{23}$-Cys$^{29}$.

**Figure 2:**
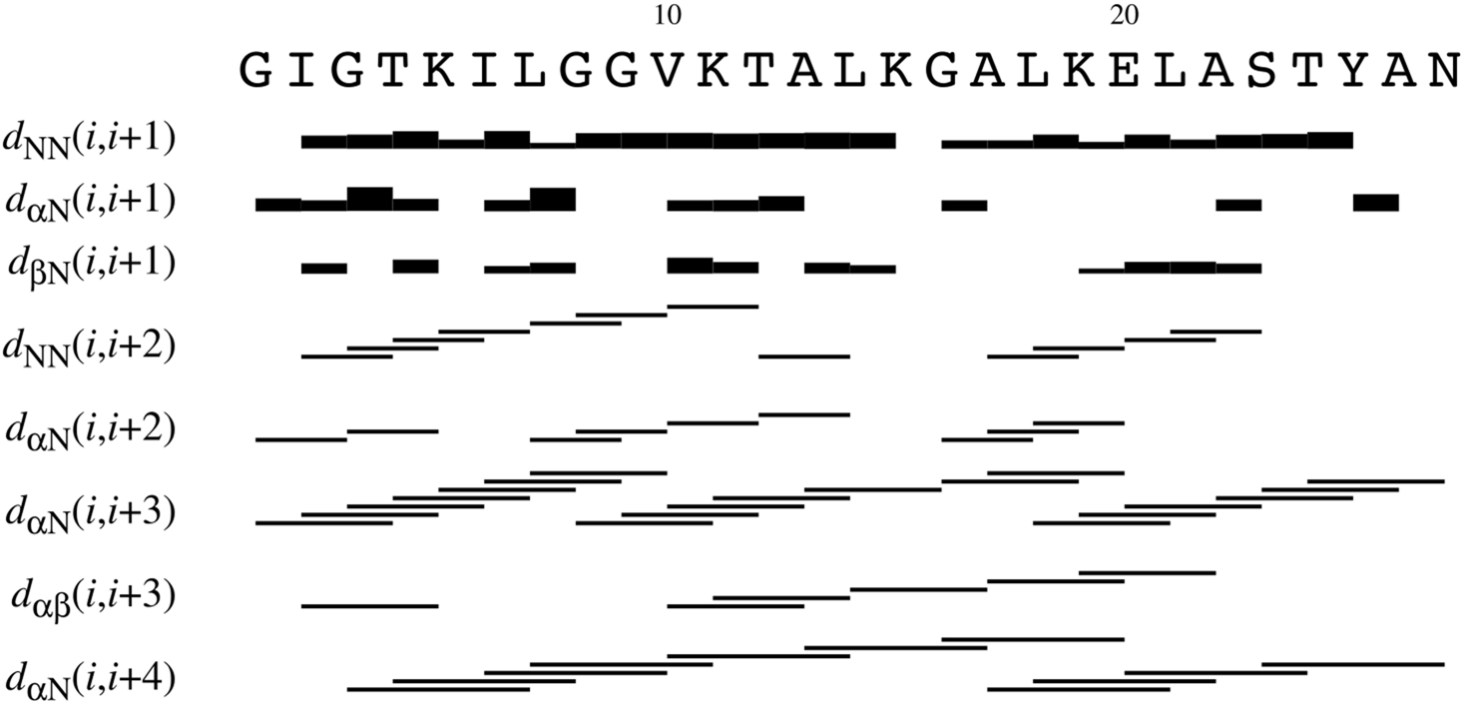
Short- and medium-range connectivities for palustrin-Ca in 50% TFE-${d_{3}}$-H$_{2}$O.

All the $^{1}$H chemical shifts identified for palustrin-Ca are detailed in Table 1. Likewise, all of palustrin-Ca’s intermolecular NOE connectivities are summarised in Fig. 2, where the NOE intensity is proportional to the thickness of each line. Multiple d$_{\alpha}$N(i, i+3), d$_{\alpha \beta }$(i, i+3), d$_{\alpha }$N(i, i+4) and d$_{\alpha \beta }$(i, i+4) were identified, which are indicative of an $\alpha$-helical secondary structure.

**Table 1:** $^{1}$H chemical shifts (ppm) identified for every residue of palustrin-Ca in 50% TFE-${d_{3}}$/H$_{2}$O.

Amino acid	NH	H$\alpha$	H$\beta$	Other protons
Gly$^{1}$		4.101, 3.923		
Phe$^{2}$	8.711	4.421	3.240, 3.075	$\delta$ 7.235; $\epsilon$ 7.321
Leu$^{3}$	8.185	4.059	1.683, 1.569	$\gamma$ 1.634; $\delta$ 0.972, 0.920
Asp$^{4}$	7.573	4.356	2.880, 2.777	
Ile$^{5}$	7.482	3.836	2.064	$\gamma$ 1.251, 0.917; $\delta$ 0.922;
Ile$^{6}$	7.761	3.731	1.970	$\gamma$1 1.482, 1.106; $\gamma$2 0.893; $\delta$ 0.808
Lys$^{7}$	8.246	4.085	1.939	$\gamma$ 1.666, 1.486; $\delta$ 1.733, $\epsilon$ 2.980
Asp$^{8}$	8.452	4.603	3.075, 2.941	
Thr$^{9}$	8.219	4.351	4.136	$\gamma$ 1.293
Gly$^{10}$	8.254	3.998		
Lys$^{11}$	7.971	4.198	2.005	$\gamma$ 1.655, 1.525; $\delta$ 1.764; $\epsilon$ 3.021, 3.005
Glu$^{12}$	8.070	4.056	2.288, 2.172	$\gamma$ 2.606, 2.457
Phe$^{13}$	8.226	4.348	3.272	$\delta$ 7.277, 7.291
Ala$^{14}$	8.014	4.035	1.587	
Val$^{15}$	8.039	3.656	2.222	$\gamma$ 1.125, 0.989
Lys$^{16}$	7.841	4.059	2.058, 1.964	$\gamma$ 1.478, 1.449; $\delta$ 1.716, 1.676; $\epsilon$ 3.007, 2.994
Ile$^{17}$	8.217	3.732	1.979	$\gamma$1 1.499, 1.071; $\gamma$2 0.888; $\delta$ 0.725
Leu$^{18}$	8.561	4.132	1.925	$\gamma$ 1.583; $\delta$ 0.922
Asn$^{19}$	8.453	4.477	2.972, 2.771	$\delta$ 6.588
Asn$^{20}$	8.063	4.521	3.051, 2.831	$\delta$ 6.789
Leu$^{21}$	8.477	4.134	1.919, 1.844	$\gamma$ 1.781; $\delta$ 0.948, 0.914
Lys$^{22}$	8.395	3.944	2.003	$\gamma$ 1.455; $\delta$ 1.720, 1.673; $\epsilon$ 2.978
Cys$^{23}$	7.987	4.328	3.472, 3.185	
Lys$^{24}$	8.200	4.185	2.091, 1.988	$\gamma$ 1.645, 1.517; $\delta$ 1.714
Leu$^{25}$	8.476	4.222	1.892	$\gamma$ 1.544; $\delta$ 0.906
Ala$^{26}$	8.040	4.471	1.546	
Gly$^{27}$	7.791	4.304, 3.936		
Gly$^{28}$	8.312	4.091, 3.936		
Cys$^{29}$	8.012	4.970	3.377, 2.951	
Pro$^{30}$		4.662	2.324, 2.064	$\gamma$ 2.006; $\delta$ 3.768, 3.661
Pro$^{31}$		4.391	2.313, 2.058	$\gamma$ 2.010; $\delta$ 3.766, 3.622

### Chemical shift analysis

**Figure 3:**
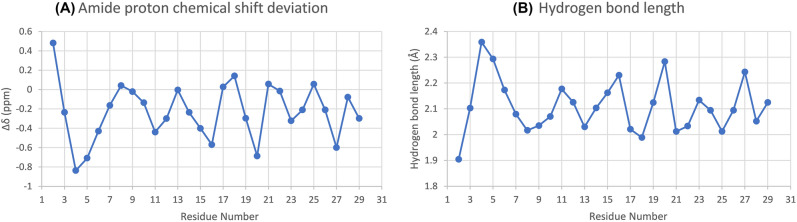
(**A**) Amide proton chemical shift deviation plot of palustrin-Ca in 50% TFE-${d_{3}}$-H$_{2}$O. The observed amide proton chemical shifts were compared to the standard random coil chemical shifts ($\Delta \delta =\delta _{obs}-\delta _{rc}$). (**B**) Hydrogen bond distance as calculated from the chemical shift deviations ($\Delta \delta = 19.2d_{N}^{-3}-2.3$), where $d_{N}$ denotes hydrogen bond length^72^.

Amide proton chemical shift deviations, calculated as the difference between the observed chemical shift and the random coil chemical shift ($\Delta \delta = \delta _{obs} - \delta _{rc}$), are connected with the hydrogen bond length^72^. Shorter hydrogen bond lengths are associated with higher $\Delta \delta$ values, and vice versa. Calculated amide proton chemical shift deviations are illustrated in Fig. 3A; the corresponding hydrogen bond lengths are given in Fig. 3B. The amide proton chemical shift deviations exhibit a periodicity of 3–5 from residue 8 on, with greater frequency in the C-terminal part of the sequence. The greatest values are found for the Phe$^{2}$, Asp$^{8}$, Phe$^{13}$, Leu$^{18}$, Leu$^{21}$, Leu$^{25}$ and Gly$^{28}$, while the lowest values are observed for Asp$^{4}$, Lys$^{11}$, Lys$^{16}$, Asn$^{20}$, Cys$^{23}$ and Gly$^{27}$. As expected, the greatest values of $\Delta \delta$ are observed for hydrophobic residues, with the exception of the Asp$^{8}$ and Gly$^{28}$ residues, which represent the beginning and end of the observed chemical shift deviation periodicity. Similarly, the lowest values are observed for polar and charged residues.

**Figure 4:**
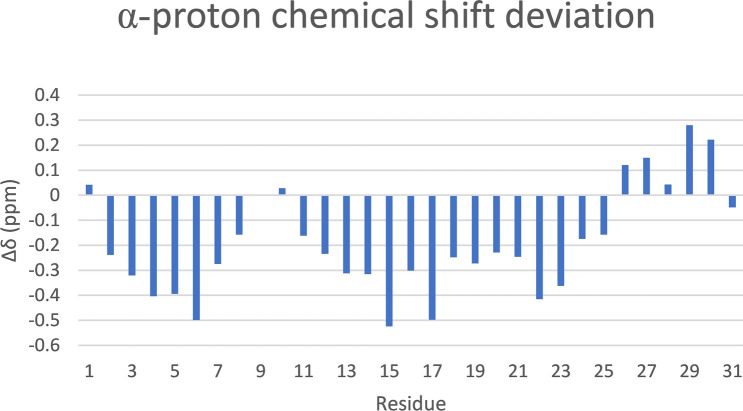
$\alpha$-proton chemical shift deviation plot of palustrin-Ca in 50% TFE-${d_{3}}$-H$_{2}$O. The observed $\alpha$-proton chemical shifts were compared to the standard random coil chemical shifts ($\Delta \delta =\delta _{obs}-\delta _{rc}$).

Similarly, $\alpha$-proton chemical shift deviations were calculated as the difference between the observed chemical shift and the random coil chemical shift ($\Delta \delta = \delta _{obs} - \delta _{rc}$). Four sequential $\alpha$-proton chemical shift deviations at least 0.1 ppm lower than the expected random coil values indicate $\alpha$-helical segments, while three or more deviations at least 0.1 ppm higher are indicative of $\beta$-strands; no change greater than 0.1 ppm is indicative of coiled regions^73^. Figure 4 shows the chemical shift deviations of palustrin-Ca, which shows a clear stretch of downfield shifts between residues Phe$^{2}$-Asp$^{8}$, and again between residues Lys$^{11}$-Leu$^{25}$. While these chemical shift deviations are indicative of $\alpha$-helical structure, the former stretch of downfield shifts may instead be attributed to a turn conformation. While the $\alpha$-proton chemical shift deviations for residues Ala$^{26}$-Pro$^{30}$ are generally upfield, this trend is not sufficiently consistent to attribute any secondary structure to it.

### Molecular modelling

**Figure 5:**
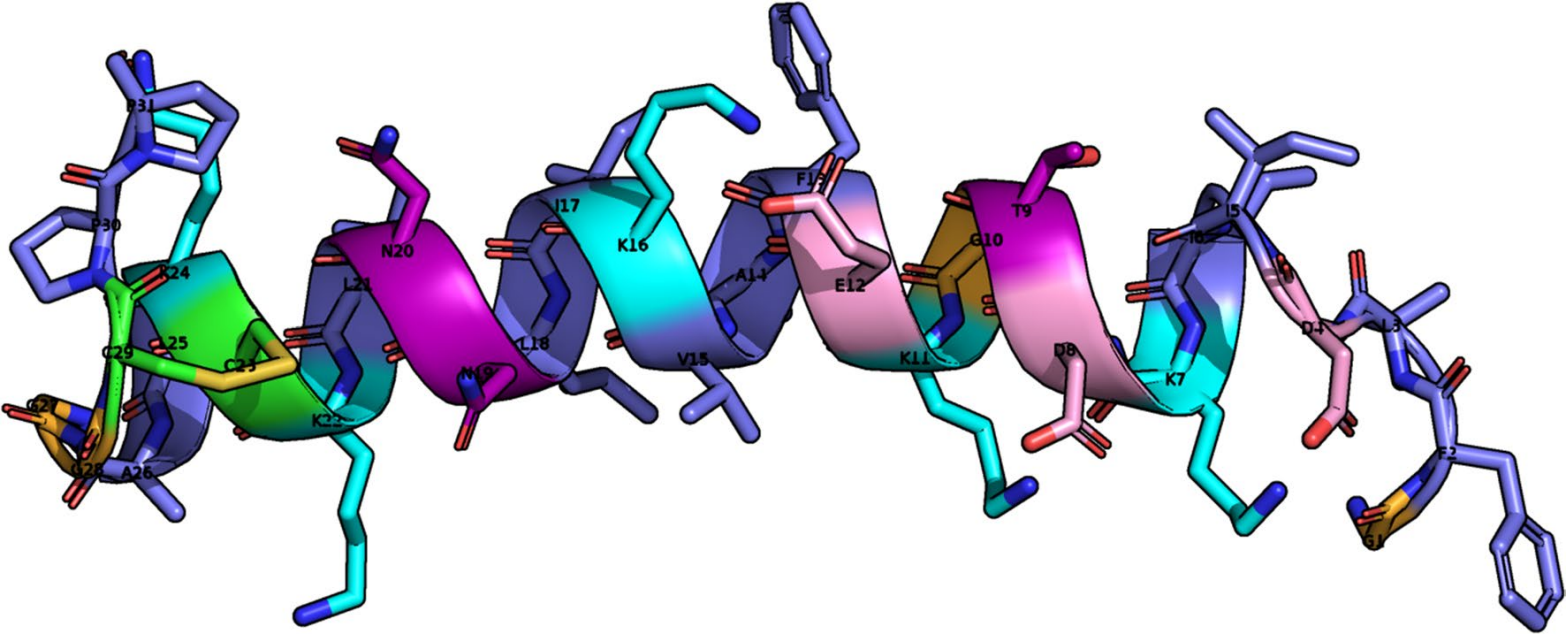
Ribbon representation of the medoid solution structure of palustrin-Ca in 50% TFE-${d_{3}}$-H$_{2}$O. The amphipathic helical motif is observed. Positively charged residues are coloured in cyan, negatively charged residues are coloured in pink, polar residues are coloured in purple, glycine residues are coloured in orange, cysteine residues are coloured in green, hydrophobic residues are coloured in slate.

The NOESY cross-peaks were integrated, and the resultant volumes were converted into distance restraints. CYANA was employed to generate one hundred structures, of which the twenty possessing the lowest target function values were further energy-minimized. Table 2 summarizes the structural and energetic statistics of the twenty selected models. As depicted in Fig. 5, palustrin-Ca is predominantly $\alpha$-helical in TFE-${d_{3}}$, which is known to promote the formation of the secondary structure. The ensemble’s secondary structural properties were analysed with the aid of STRIDE^74^. Most of the ensemble’s structures are display $\alpha$-helical structures between residues Ile$^{6}$-Ala$^{26}$. The terminal segments are predominantly defined as turns, but possess significant coil character as well. More specifically, the N-terminus exhibits mostly turn character, although the coil character of the C-terminus is greater. The peptide structure is amphipathic, with a clear separation between the polar and non-polar faces of the helix. This is unsurprising, considering that amphipathicity is a common requirement for antimicrobial activity^75^.

**Table 2:** Mean structural statistics of the twenty structural models comprising the palustrin-Ca ensemble.

**NOEs**	
Intraresidue	147
Interresidue, sequential	144
Interresidue, nonsequential	130
**Ensemble RMSD values, Å, SA ± SD**^**a**^	
**Backbone**	
All residues	1.270 ± 0.418
N-terminal segment (G1-I5)	1.624 ± 0.534
Helix (I6-A26)	0.754 ± 0.181
C-terminal segment (G27-P31)	2.142 ± 1.032
**Heavy atoms**	
All residues	1.638 ± 0.359
N-terminal segment (G1-I5)	2.073 ± 0.447
Helix (I6-A26)	1.279 ± 0.190
C-terminal segment (G27-P31)	2.397 ± 1.175
**Ramachandran plot analysis**^b^	
Residues in most favoured regions	84.2%
Residues in additionally allowed regions	9.6%
Residues in generously allowed regions	6.2%
Residues in disallowed regions	0%
**Average energies (kcal/mol)**	
E$_\text{bond}$	22.952
E$_\text{angle}$	134.653
E$_\text{dihed}$	91.179
E$_\text{imprp}$	5.888
E$_\text{VdW}$	− 42.819
E$_\text{elec}$	− 501.612
E$_\text{total}$	− 292.328

The peptide structure has been deposited in the PDB with deposition code 7P4X. Model 10 is the representative medoid structure.

^a^RMSD values from VMD.

^b^Based on PROCHECK.

### Molecular dynamics

**Figure 6:**
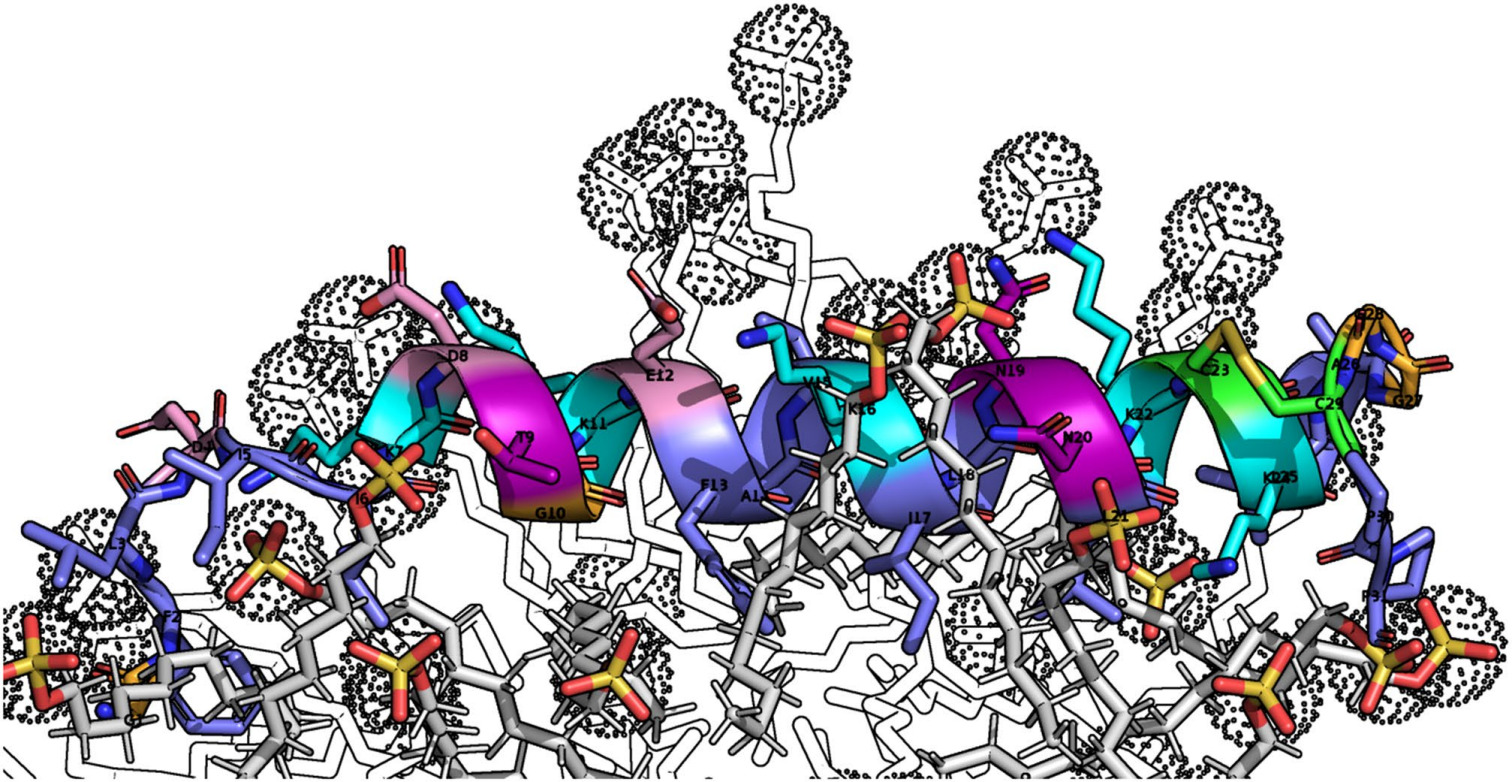
Palustrin-Ca positioned at the SDS micelle-water boundary. Lysine residue side chains are shown interacting with the negatively charged sulfate headgroups. Positively charged residues are coloured in cyan, negatively charged residues are coloured in pink, polar residues are coloured in purple, glycine residues are coloured in orange, cysteine residues are coloured in green, hydrophobic residues are coloured in slate. SDS aliphatic chains are coloured in white, while the SDS headgroups are orange and red, and highlighted with dotted spheres.

A molecular dynamics simulation of palustrin-Ca’s medoid structure in an SDS micelle was conducted to characterise its behaviour in the prokaryotic membrane-mimetic environment. SDS micelles are often used as mimetics of the bacterial membrane in molecular dynamics simulations^76–80^. In the course of the simulation, the peptide translocated from its initial position aligned with the micelle’s centre of mass, to the micelle’s surface-water boundary, where it adopted a position parallel to the micelle surface (Fig. 6). The peptide’s amphipathicity is clearly apparent; its hydrophobic residues remain closely associated with the micellar interior, while the hydrophilic residues are in contact with the aqueous solvent and the micelle’s anionic sulfate headgroups. This amphipathic configuration is quite energetically favourable, and is contributes to the peptide’s exertion of its antimicrobial activity^81^. The peptide’s cationic residues are important for its bioactivity, as the electrostatic interaction between the target surface’s anionic charges and a peptide’s cationic residues drives the initial attraction of the peptide to the target^82^. Likewise, the hydrophobic residues are necessary for the peptide’s insertion into the target hydrophobic core^83^.

**Figure 7:**
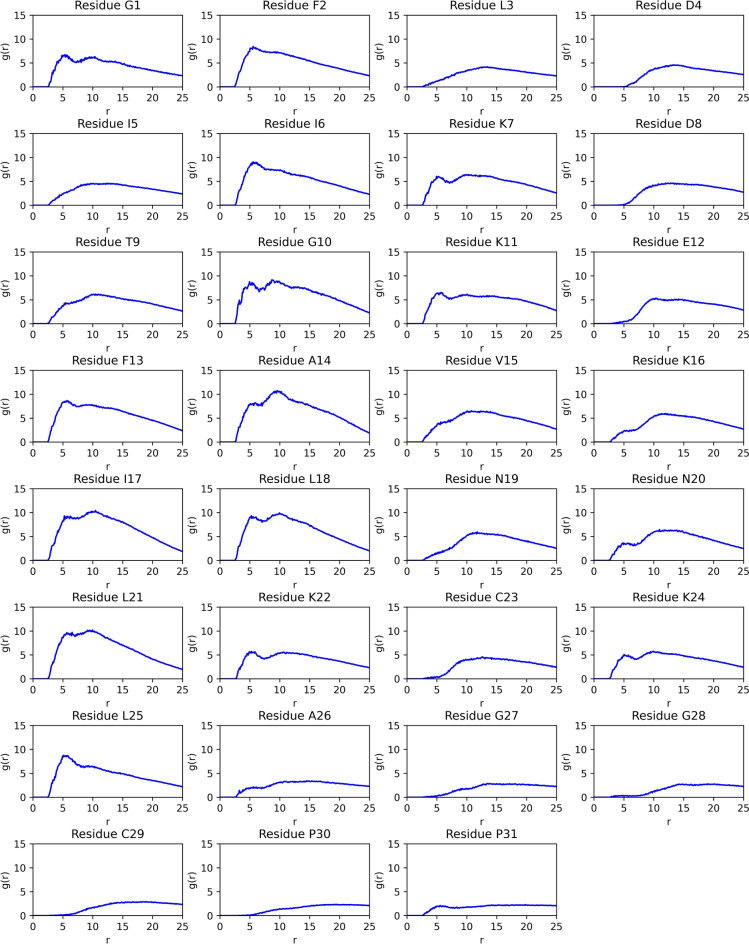
Radial distribution function (RDF) *g(r)* plotted between palustrin-Ca’s residues’ carbon atoms and the sodium dodecyl sulfate (SDS) micelle aliphatic chain.

The peptide’s interactions with the micelle’s hydrophobic core were quantified using the radial distribution function *g(r)*, which was calculated between the peptide’s carbon atoms and the micelle’s aliphatic chain, and plotted against the radius *r*, as shown in Fig. 7.

The radial distribution function plots illustrate clearly that the peptide’s hydrophobic residues maintain a close association with the micelle, especially in the structured $\alpha$-helical segment, where the hydrophobic Ile$^{6}$, Phe$^{13}$, Ala$^{14}$, Ile$^{17}$, Leu$^{18}$, Leu$^{21}$ and Leu$^{25}$ residues are particularly closely associated to the micelle hydrophobic core. Conversely, hydrophilic residues, including Asp$^{8}$, Glu$^{12}$, Lys$^{16}$, Asn$^{19}$ and Lys$^{22}$ are less associated with the micelle core, and in close contact with the aqueous solvent.

Interestingly, the N-terminal segment of the peptide is also found to maintain close association with the micelle, especially through the Gly$^{1}$, Phe$^{2}$ and Ile$^{6}$ residues, despite the first five residues not forming part of a defined secondary structure. This is in contrast to the C-terminal segment, where beginning with Ala$^{26}$ onwards the peptide is only weakly associated with the micelle.

**Figure 8:**
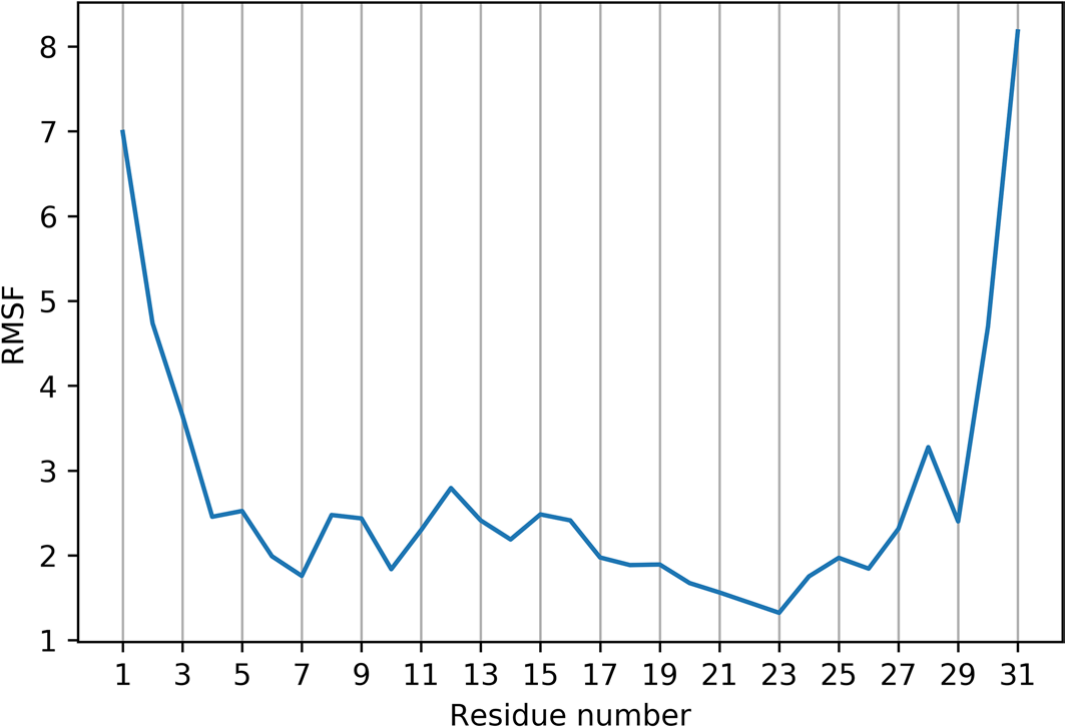
Root-mean-square fluctuation (RMSF) calculated for the backbone atoms of each residue of palustrin-Ca over the course of the simulation. It is apparent that the peptide structure is generally rigid, with the exception of the N- and C-terminal residues, which are relatively flexible.

The peptide, which was experimentally determined to be $\alpha$-helical between residues Ile$^{6}$ and Ala$^{26}$, maintains this structure throughout the duration of the simulation. Despite the presence of a glycine residue at position 10, the $\alpha$-helix remains relatively rigid, not exhibiting any significant flexibility during the simulation. This suggests that the glycine residue occurs too far from the helical segment’s middle to impart flexibility. Although studies have previously demonstrated that a rigid $\alpha$-helical structure can potentially result in an increased haemolytic activity^84^, this does not appear to apply in this case, which may be explained by the relatively flexible non-helical N-terminal and C-terminal segments, which combined account for 10 of the sequences 31 residues. This flexibility can be seen in the root-mean-square fluctuation (RMSF) plot, where the peptide’s terminal residues have greater RMSF values than the residues located within the $\alpha$-helical segment (Fig. 8).

## Conclusion

A number of biologically active peptides have been isolated from the American bullfrog *Lithobates catesbeianus*. The isolated peptides are diverse, and not limited to a single peptide family; palustrin-Ca is the only palustrin peptide isolated from this amphibian. Palustrin-Ca can be considered to be a member of the palustrin-2 family of peptides, sharing an average 42.09% sequence identity with 18 peptides of the palustrin-2 family. Peptides that belong to the palustrin-2 family exhibit a variety of potent biological activities, including antimicrobial and anticancer activities, and therefore represent exciting candidates for novel drug development.

Palustrin-Ca is 31 amino acid residues long, of which 5 are lysine, 4 are glycine and 4 are leucine. The peptide has a cyclic disulfide-bridged heptapeptide domain at its C-terminus, which is conserved with other peptides of the palustrin-2 family.

This study employed NMR spectroscopy and molecular modelling methods to characterise the peptide’s three-dimensional structure and obtain an ensemble of model structures. To date, palustrin-Ca is the only palustrin peptide to have had its three-dimensional structure elucidated. The results established that palustrin-Ca possesses an amphipathic, $\alpha$-helical structure between residues Ile$^{6}$-Ala$^{26}$ in a 50% TFE-H$_{2}$O mixed solvent system, with the terminal segments predominantly defined as turns, with significant coil character as well.

A molecular dynamics simulation was conducted, whereby the medoid NMR-determined structure was simulated with an SDS micelle. The simulation results suggest that palustrin-Ca preferentially adopts a position parallel to the micelle’s surface. This configuration is most energetically favourable as the peptide’s hydrophobic residues can penetrate to the micelle’s hydrophobic core, while the hydrophilic residues remain in contact with the aqueous solvent. The RDF plot in Fig. 7 illustrates this clearly; the greatest values for *g*(*r*) at small distances are observed for the hydrophobic Leu, Ile, Ala and Phe residues, demonstrating the importance of hydrophobicity for a peptide’s ability to bind the target membrane. The observed preference for a position parallel to the micelle surface indicates that the peptide most probably exerts its antimicrobial activity through a non-pore-forming mechanism of action, such as the carpet model or the interfacial activity model.

This study’s results will facilitate future work focused on investigating HDP structures, and their interactions with zwitterionic lipid bilayers, in order to further our understanding of the relationships between HDP structure and function. This study has extended earlier work from our research group that led to the elucidation of a number of host-defence peptide structures, and also complements related bioinformatic studies on peptide structure and function^85–88^.

## Supplementary Information

Supplementary Information.

## References

[1] Xia X, Cheng L, Zhang S, Wang L, Hu J (2018). The role of natural antimicrobial peptides during infection and chronic inflammation. Antonie Van Leeuwenhoek.

[2] Maróti Gergely G, Kereszt A, Kondorosi É, Mergaert P (2011). Natural roles of antimicrobial peptides in microbes, plants and animals. Res. Microbiol..

[3] Avila EE (2017). Functions of antimicrobial peptides in vertebrates. Curr. Protein Pept. Sci..

[4] da Silva Pereira L (2018). Characterization of *Capsicum annuum* L. leaf and root antimicrobial peptides: Antimicrobial activity against phytopathogenic microorganisms. Acta Physiologiae Plantarum.

[5] El Samak M, Solyman SM, Hanora A (2018). Antimicrobial activity of bacteria isolated from Red Sea marine invertebrates. Biotechnol. Rep..

[6] Gordon YJ, Romanowski EG, McDermott AM (2005). Mini review: A review of antimicrobial peptides and their therapeutic potential as anti-infective drugs. Curr. Eye Res..

[7] Tanphaichitr N (2016). Potential use of antimicrobial peptides as vaginal spermicides/microbicides. Pharmaceuticals.

[8] Gaspar D, Salomé Veiga A, Castanho MA (2013). From antimicrobial to anticancer peptides. A review. Front. Microbiol..

[9] Agarwal G, Gabrani R (2021). Antiviral Peptides: Identification and Validation. Int. J. Pept. Res. Ther..

[10] Lacerda AF, Pelegrini PB, De Oliveira DM, Vasconcelos ÉA, Grossi-de Sá MF (2016). Anti-parasitic peptides from arthropods and their application in drug therapy. Front. Microbiol..

[11] Zhao, R.-L., Han, J.-Y., Han, W.-Y., He, H.-X. & Ma, J.-F. Effects of two novel peptides from skin of lithobates catesbeianus on tumor cell morphology and proliferation. In *Molecular Cloning—Selected Applications in Medicine and Biology* (InTech, 2011). http://aps.unmc.edu/AP.

[12] Lai R, Liu H, Hui Lee W, Zhang Y (2002). An anionic antimicrobial peptide from toad Bombina maxima. Biochem. Biophys. Res. Commun..

[13] Zelezetsky I, Tossi A (2006). Alpha-helical antimicrobial peptides—Using a sequence template to guide structure–activity relationship studies. Biochim. Biophys. Acta Biomembr..

[14] Xie J (2017). Novel antimicrobial peptide CPF-C1 analogs with superior stabilities and activities against multidrug-resistant bacteria. Chem. Biol. Drug Des..

[15] Yin LM, Edwards MA, Li J, Yip CM, Deber CM (2012). Roles of hydrophobicity and charge distribution of cationic antimicrobial peptides in peptide-membrane interactions. J. Biol. Chem..

[16] Son M, Lee Y, Hwang H, Hyun S, Yu J (2013). Disruption of interactions between hydrophobic residues on nonpolar faces is a key determinant in decreasing hemolysis and increasing antimicrobial activities of $$\alpha$$-helical amphipathic peptides. ChemMedChem.

[17] Hornef MW, Pütsep K, Karlsson J, Refai E, Andersson M (2004). Increased diversity of intestinal antimicrobial peptides by covalent dimer formation. Nat. Immunol..

[18] Lorenzon EN, Piccoli JP, Santos-Filho NA, Cilli EM (2019). Dimerization of antimicrobial peptides: A promising strategy to enhance antimicrobial peptide activity. Protein Pept. Lett..

[19] Beveridge TJ (1999). Structures of gram-negative cell walls and their derived membrane vesicles. J. Bacteriol..

[20] Weidenmaier C, Peschel A (2008). Teichoic acids and related cell-wall glycopolymers in Gram-positive physiology and host interactions. Nat. Rev. Microbiol..

[21] Zanin LMP (2013). Interaction of a synthetic antimicrobial peptide with model membrane by fluorescence spectroscopy. Eur. Biophys. J..

[22] Hong SY, Park TG, Lee KH (2001). The effect of charge increase on the specificity and activity of a short antimicrobial peptide. Peptides.

[23] Yeaman MR, Yount NY (2003). Mechanisms of antimicrobial peptide action and resistance. Pharmacol. Rev..

[24] Perron GG, Zasloff M, Bell G (2006). Experimental evolution of resistance to an antimicrobial peptide. Proc. R. Soc. B Biol. Sci..

[25] Hall K, Lee TH, Mechler AI, Swann MJ, Aguilar MI (2014). Real-time measurement of membrane conformational states induced by antimicrobial peptides: Balance between recovery and lysis. Sci. Rep..

[26] Sani MA, Henriques ST, Weber D, Separovic F (2015). Bacteria may cope differently from similar membrane damage caused by the Australian tree frog antimicrobial peptide maculatin 1.1. J. Biol. Chem..

[27] Brogden KA (2005). Antimicrobial peptides: Pore formers or metabolic inhibitors in bacteria?. Nat. Rev. Microbiol..

[28] Shai Y (2002). Mode of action of membrane active antimicrobial peptides. Pept. Sci. Orig. Res. Biomol..

[29] Hammond K (2021). Switching cytolytic nanopores into antimicrobial fractal ruptures by a single side chain mutation. ACS Nano.

[30] Sani MA, Separovic F (2016). How membrane-active peptides get into lipid membranes. Acc. Chem. Res..

[31] Subasinghage AP, Conlon JM, Hewage CM (2008). Conformational analysis of the broad-spectrum antibacterial peptide, ranatuerin-2CSa: Identification of a full length helix-turn-helix motif. Biochim. Biophys. Acta Proteins Proteomics.

[32] Subasinghage AP, Conlon JM, Hewage CM (2010). Development of potent anti-infective agents from *Silurana tropicalis*: Conformational analysis of the amphipathic, alpha-helical antimicrobial peptide XT-7 and its non-haemolytic analogue [G4K]XT-7. Biochim. Biophys. Acta Proteins Proteomics.

[33] Subasinghage AP, O’Flynn D, Conlon JM, Hewage CM (2011). Conformational and membrane interaction studies of the antimicrobial peptide alyteserin-1c and its analogue [E4K]alyteserin-1c. Biochim. Biophys. Acta Biomembr..

[34] Timmons PB, O’Flynn D, Conlon JM, Hewage CM (2019). Structural and positional studies of the antimicrobial peptide brevinin-1BYa in membrane-mimetic environments. J. Pept. Sci..

[35] Timmons PB, O’Flynn D, Conlon JM, Hewage CM (2019). Insights into conformation and membrane interactions of the acyclic and dicarba-bridged brevinin-1BYa antimicrobial peptides. Eur. Biophys. J..

[36] Benetti S, Timmons PB, Hewage CM (2019). NMR model structure of the antimicrobial peptide maximin 3. Eur. Biophys. J..

[37] Timmons PB, Hewage CM (2021). Biophysical study of the structure and dynamics of the antimicrobial peptide maximin 1. J. Pept. Sci..

[38] Bax A, Davis DG (1985). MLEV-17-based two-dimensional homonuclear magnetization transfer spectroscopy. J. Magn. Resonance (1969).

[39] Kumar A, Ernst RR, Wüthrich K (1980). A two-dimensional nuclear Overhauser enhancement (2D NOE) experiment for the elucidation of complete proton-proton cross-relaxation networks in biological macromolecules. Top. Catal..

[40] John BK, Plant D, Webb P, Hurd RE (1992). Effective combination of gradients and crafted RF pulses for water suppression in biological samples. J. Magn. Resonance.

[41] Lee W, Tonelli M, Markley JL (2015). NMRFAM-SPARKY: Enhanced software for biomolecular NMR spectroscopy. Bioinformatics.

[42] Güntert P, Braun W, Wüthrich K (1991). Efficient computation of three-dimensional protein structures in solution from nuclear magnetic resonance data using the program DIANA and the supporting programs CALIBA, HABAS and GLOMSA. J. Mol. Biol..

[43] Güntert, P., Mumenthaler, C. & Wüthrich, K. Torsion angle dynamics for NMR structure calculation with the new program DYANA. *J. Mol. Biol.* **273**, 283–298 (1997). 10.1006/jmbi.1997.1284 9367762

[44] Güntert P, Buchner L (2015). Combined automated NOE assignment and structure calculation with CYANA. J. Biomol. NMR.

[45] Mackerell AD, Feig M, Brooks CL (2004). Extending the treatment of backbone energetics in protein force fields: Limitations of gas-phase quantum mechanics in reproducing protein conformational distributions in molecular dynamics simulation. J. Comput. Chem..

[46] Phillips JC (2005). Scalable molecular dynamics with NAMD. J. Comput. Chem..

[47] Humphrey W, Dalke A, Schulten KVMD (1996). Visual molecular dynamics. J. Mol. Graph..

[48] Laskowski RA, MacArthur MW, Moss DS, Thornton JM (1993). PROCHECK: A program to check the stereochemical quality of protein structures. J. Appl. Crystallogr..

[49] Berman H, Henrick K, Nakamura H (2003). Announcing the worldwide Protein Data Bank. Nat. Struct. Biol..

[50] Jakobtorweihen S, Ingram T, Smirnova I (2013). Combination of COSMOmic and molecular dynamics simulations for the calculation of membrane-water partition coefficients. J. Comput. Chem..

[51] MacKerell AD (1998). All-atom empirical potential for molecular modeling and dynamics studies of proteins. J. Phys. Chem. B.

[52] Martyna GJ, Tobias DJ, Klein ML (1994). Constant pressure molecular dynamics algorithms. J. Chem. Phys..

[53] Feller SE, Zhang Y, Pastor RW, Brooks BR (1995). Constant pressure molecular dynamics simulation: The Langevin piston method. J. Chem. Phys..

[54] Darden T, York D, Pedersen L (1993). Particle mesh Ewald: An N$$\cdot $$log(N) method for Ewald sums in large systems. J. Chem. Phys..

[55] Ryckaert JP, Ciccotti G, Berendsen HJ (1977). Numerical integration of the cartesian equations of motion of a system with constraints: Molecular dynamics of n-alkanes. J. Comput. Phys..

[56] Sönnichsen FD, Van Eyk JE, Hodges RS, Sykes BD (1992). Effect of trifluoroethanol on protein secondary structure: An NMR and CD study using a synthetic actin peptide. Biochemistry.

[57] Goodman M, Rosen IG (1964). Conformational aspects of polypeptide structure XVI. Rotatory constants, cotton effects, and ultraviolet absorption data for glutamate oligomers and co-oligomers. Biopolymers.

[58] Buck M (1998). Trifluoroethanol and colleagues: Cosolvents come of age. Recent studies with peptides and proteins. Q. Rev. Biophys..

[59] Bodkin MJ, Goodfellow JM (1996). Hydrophobic solvation in aqueous trifluoroethanol solution. Biopolymers.

[60] Yi GS, Park CB, Kim SC, Cheong C (1996). Solution structure of an antimicrobial peptide buforin II. FEBS Letters.

[61] Seo MD, Won HS, Kim JH, Mishig-Ochir T, Lee BJ (2012). Antimicrobial peptides for therapeutic applications: A review. Molecules.

[62] Raj PA, Marcus E, Sukumaran DK (1998). Structure of human salivary histatin 5 in aqueous and nonaqueous solutions. Biopolymers.

[63] Gong Z, Ikonomova SP, Karlsson AJ (2018). Secondary structure of cell-penetrating peptides during interaction with fungal cells. Protein Sci..

[64] Pirtskhalava M, Vishnepolsky B, Grigolava M, Managadze G (2021). Physicochemical features and peculiarities of interaction of amp with the membrane. Pharmaceuticals.

[65] Shiraki K, Nishikawa K, Goto Y (1995). Trifluoroethanol-induced stabilization of the $$\alpha$$-helical structure of $$\beta$$-lactoglobulin: Implication for non-hierarchical protein folding. J. Mol. Biol..

[66] Venneti KC, Hewage CM (2011). Conformational and molecular interaction studies of glucagon-like peptide-2 with its N-terminal extracellular receptor domain. FEBS Lett..

[67] Rajan R, Balaram P (1996). A model for the interaction of trifluoroethanol with peptides and proteins. Int. J. Pept. Protein Res..

[68] Roccatano D, Colombo G, Fioroni M, Mark AE (2002). Mechanism by which 2,2,2-trifluoroethanol/water mixtures stabilize secondary-structure formation in peptides: A molecular dynamics study. Proc. Natl. Acad. Sci. USA.

[69] Marion D, Zasloff M, Bax A (1988). A two-dimensional NMR study of the antimicrobial peptide magainin 2. FEBS Lett..

[70] Toke O (2011). A kinked antimicrobial peptide from Bombina maxima. I. Three-dimensional structure determined by NMR in membrane-mimicking environments. Eur. Biophys. J..

[71] Alaña I, Malthouse JPG, O’Harte FP, Hewage CM (2007). The bioactive conformation of glucose-dependent insulinotropic polypeptide by NMR and CD spectroscopy. Proteins Struct. Funct. Genet..

[72] Wagner G, Pardi A, Wüthrich K (1983). Hydrogen bond length and 1H NMR chemical shifts in proteins. J. Am. Chem. Soc..

[73] Wishart DS, Sykes BD, Richards FM (1992). The chemical shift index: A fast and simple method for the assignment of protein secondary structure through NMR spectroscopy. Biochemistry.

[74] Frishman D, Argos P (1995). Knowledge-based protein secondary structure assignment. Proteins Struct. Funct. Bioinform..

[75] Tossi A, Sandri L, Giangaspero A (2000). Amphipathic, $$\alpha$$-helical antimicrobial peptides. Biopolymers.

[76] Langham A, Kaznessis YN (2010). Molecular simulations of antimicrobial peptides. Methods Mol. Biol..

[77] Khandelia H, Langham AA, Kaznessis YN (2006). Driving engineering of novel antimicrobial peptides from simulations of peptide-micelle interactions. Biochim. Biophys. Acta Biomembr..

[78] Clark TD, Bartolotti L, Hicks RP (2013). The application of DOSY NMR and molecular dynamics simulations to explore the mechanism(s) of micelle binding of antimicrobial peptides containing unnatural amino acids. Biopolymers.

[79] Roussel G (2018). Peptide-surfactant interactions: A combined spectroscopic and molecular dynamics simulation approach. Spectrochim. Acta Part A Mol. Biomol. Spectrosc..

[80] Crusca E (2017). NMR structures and molecular dynamics simulation of hylin-a1 peptide analogs interacting with micelles. J. Pept. Sci..

[81] Dathe M, Wieprecht T (1999). Structural features of helical antimicrobial peptides: Their potential to modulate activity on model membranes and biological cells. Biochim. Biophys. Acta Biomembr..

[82] Shai Y, Oren Z (2001). From, “carpet” mechanism to de-novo designed diastereomeric cell-selective antimicrobial peptides. Peptides.

[83] Chen Y (2007). Role of peptide hydrophobicity in the mechanism of action of $$\alpha$$-helical antimicrobial peptides. Antimicrob. Agents Chemother..

[84] Idiong G (2011). Investigating the effect of a single glycine to alanine substitution on interactions of antimicrobial peptide latarcin 2a with a lipid membrane. Eur. Biophys. J.

[85] Timmons PB, Hewage CM (2020). HAPPENN is a novel tool for hemolytic activity prediction for therapeutic peptides which employs neural networks. Sci. Rep..

[86] Timmons PB, Hewage CM (2021). ENNAACT is a novel tool which employs neural networks for anticancer activity classification for therapeutic peptides. Biomed. Pharmacother..

[87] Timmons PB, Hewage CM (2021). ENNAVIA is a novel method which employs neural networks for antiviral and anti-coronavirus activity prediction for therapeutic peptides. Brief. Bioinform..

[88] Timmons PB, Hewage CM (2021). APPTEST is a novel protocol for the automatic prediction of peptide tertiary structures. Brief. Bioinform..

