# Supplementary material for: Conformation and membrane interaction studies of the potent antimicrobial and anticancer peptide palustrin-Ca
Source: Sci Rep. 2021 Nov 17;11:22468. doi: 10.1038/s41598-021-01769-3 (PMC8599514; doi:10.1038/s41598-021-01769-3)

Conformation and membrane interaction studies of the potent antimicrobial and anticancer peptide palustrin-Ca

Supplementary Information

Mass Spectrometry

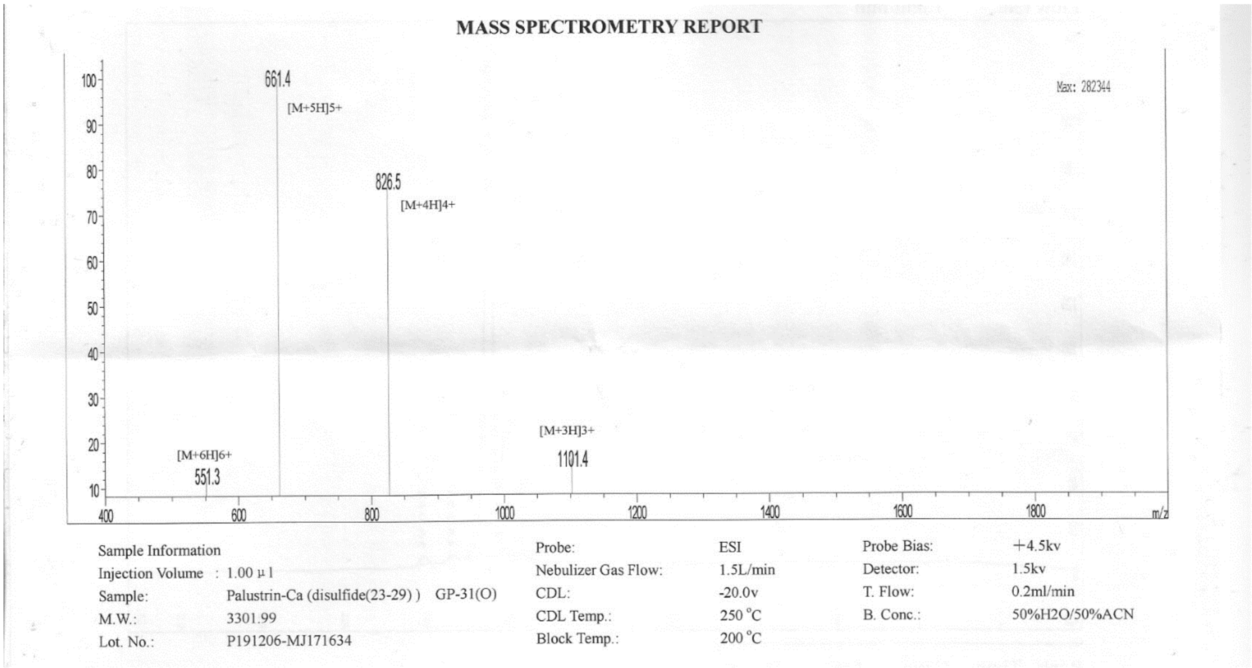

HPLC

Sample: Palustrin-Ca (disulfide (23-29)) GP-31(0)

Lot. No.: P191206-MJ171634

Column: Kromasil 100-5C18, 4.6*250mm, 5μm

Solvent A A: 0.1% Trifluoroacetic Acid in 100% Acetonitrile

Solvent B B: 0.1% Trifluoroacetic Acid in 100% Water

Gradient:

	A	B
0.0min	29%	71%
25.0min	54%	46%
25.1min	100%	0%
30.0min	Stop	

Volume: 10μl

Wavelength: 220nm

Flow rate: 1.0ml/min

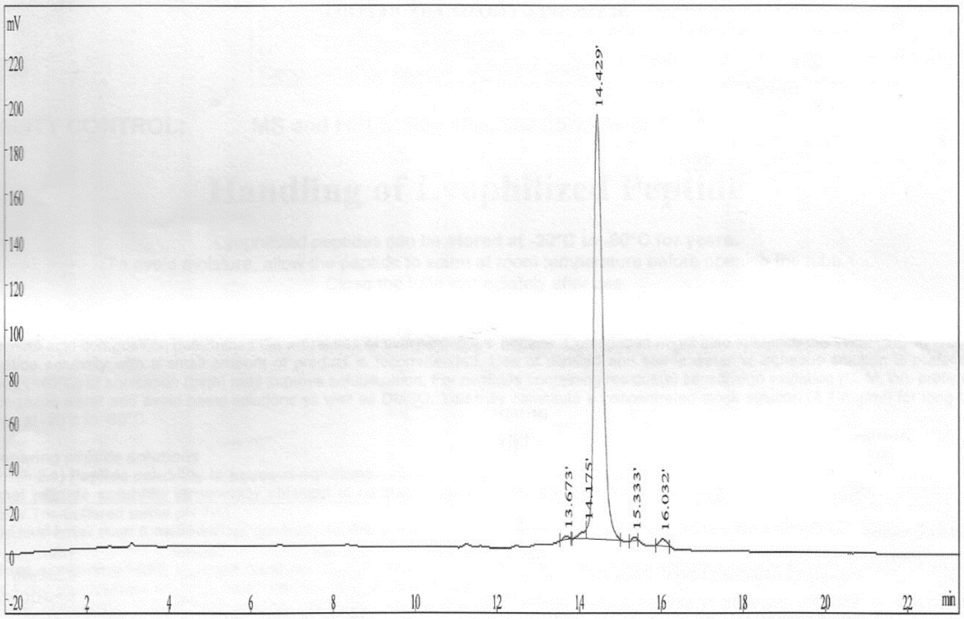

Supplement: Supplementary file 1 — Supplementary Information. [file 41598_2021_1769_MOESM1_ESM.pdf]
